# Enforced expression of phosphatidylinositol 4-phosphate 5-kinase homolog alters PtdIns(4,5)P_2_ distribution and the localization of small G-proteins

**DOI:** 10.1038/s41598-019-51272-z

**Published:** 2019-10-15

**Authors:** Yanbo Yang, Miriam Park, Masashi Maekawa, Gregory D. Fairn

**Affiliations:** 1 Department of Biochemistry, University of Toronto, ON M5S 1A8 Toronto, Canada; 2 Keenan Research Centre for Biomedical Sciences, St. Michael’s Hospital, 209 Victoria Street, Toronto, ON M5B 1T8 Canada; 3 Division of Cell Growth and Tumor Regulation, Proteo-Science Center, Ehime University, Ehime, Japan; 4 Department of Biochemistry and Molecular Genetics, Ehime University Graduate School of Medicine, Ehime, Japan; 5 Department of Surgery, University of Toronto, Toronto, ON M5T 1P5 Canada

**Keywords:** Lipids, Membrane trafficking

## Abstract

The generation of phosphatidylinositol 4,5-bisphosphate (PtdIns(4,5)P_2_) by phosphatidylinositol 4-phosphate 5-kinases (PIP5Ks) is essential for many functions including control of the cytoskeleton, signal transduction, and endocytosis. Due to its presence in the plasma membrane and anionic charge, PtdIns(4,5)P_2_, together with phosphatidylserine, provide the inner leaflet of the plasma membrane with a negative surface charge. This negative charge helps to define the identity of the plasma membrane, as it serves to recruit or regulate a multitude of peripheral and membrane proteins that contain polybasic domains or patches. Here, we determine that the phosphatidylinositol 4-phosphate 5-kinase homolog (PIPKH) alters the subcellular distribution of PtdIns(4,5)P_2_ by re-localizing the three PIP5Ks to endomembranes. We find a redistribution of the PIP5K family members to endomembrane structures upon PIPKH overexpression that is accompanied by accumulation of PtdIns(4,5)P_2_ and phosphatidylinositol 3,4,5-trisphosphate (PtdIns(3,4,5)P_3_). PIP5Ks are targeted to membranes in part due to electrostatic interactions; however, the interaction between PIPKH and PIP5K is maintained following hydrolysis of PtdIns(4,5)P_2_. Expression of PIPKH did not impair bulk endocytosis as monitored by FM4-64 uptake but did result in clustering of FM4-64 positive endosomes. Finally, we demonstrate that accumulation of polyphosphoinositides increases the negative surface charge of endosomes and in turn, leads to relocalization of surface charge probes as well as the polycationic proteins K-Ras and Rac1.

## Introduction

Phosphoinositides (PIPs), the phosphorylated derivatives of phosphatidylinositol, constitute a relatively minor fraction of the total cellular lipids^1^. For the most part, PIPs are synthesized locally on organelles by specific kinases and are subject to degradation or consumption by a variety of phosphatases and phospholipase C isoforms^1^. The inositol ring has three accessible hydroxyl groups that by differential phosphorylation lead to the generation of seven PIP species^1,2^. Many of the individual PIPs display preferential accumulation on specific organelles, and thus the presence of specific PIP species has been postulated to provide identity to its organelle^2^.

Another biophysical feature of organellar membranes that contributes to their identity is the negative surface charge of their cytosolic leaflets. Experimental evidence has demonstrated that the inner leaflet of the plasma membrane possesses the highest negative charge density of the organelles^3–5^. This membrane is not only rich in the anionic phospholipids phosphatidylserine and phosphatidylinositol but also PIPs including PtdIns(4)P and PtdIns(4,5)P_2_^6^. The negative surface charge of the inner leaflet of the plasma membrane along with PtdIns(4,5)P_2_ and PtdIns(3,4,5)P_3_ target numerous peripheral proteins to the plasma membrane via either electrostatic interactions or by serving as ligands for modular protein domains^7,8^. Indeed, many peripheral proteins target the plasma membrane via a coincidence sensing typically through phospholipid binding and lipid modification such as prenylation or a polybasic patch^9,10^.

Mammalian cells produce PtdIns(4,5)P_2_ using two related yet distinct mechanisms. Type I phosphatidylinositol 4-phosphate 5-kinases (PIP5Ks) are present throughout the eukaryotic kingdom and are responsible for the bulk of PtdIns(4,5)P_2_ synthesis^11,12^. The second pathway that synthesizes PtdIns(4,5)P_2_ is mediated by type II phosphatidylinositol 5-phosphate 4-kinases that use PtdIns(5)P as a substrate^12^. The type I and II enzymes appear to have arisen from a common ancestor and display ≈30% sequence identity^13^. Despite this similarity, the type I and type II kinases localize to different subcellular locations^6,14^. Previous results demonstrated that the plasmalemmal localization of the type I kinases is due to the presence of its substrate PtdIns(4)P and the anionic surface charge of the inner leaflet of the plasma membrane^6^. Curiously, swapping of the activation loop of the type I and type II kinases is sufficient to switch not only their substrate preference but also their subcellular localization^15^. Initial structural studies suggested that the activation loop lacked structure; however, recent NMR studies suggest that adopts an alpha-helical structure following interactions with phosphatidylcholine containing micelles^16^. It is also worth considering that the mechanisms used to target PIP5K to membranes are potentiated by the ability of the individual isoforms to dimerize or form heterodimers (e.g., β-γ heterodimer)^17,18^. Indeed, recent determination of the crystal structure of zebrafish PIP5Kα demonstrated that this protein forms a side-by-side dimer with both the N- and C-terminal lobes of the protein contributing to the formation of the dimer^18^. Finally, in addition to substrate recognition and electrostatic charge, type I kinases are reported to interact with several proteins that could influence their subcellular distribution including the small G-proteins Arf6, Rho and Bruton’s Tyrosine Kinase^19–21^. Currently, the impact of protein-protein interactions on the activity and subcellular localization of PIP5Ks is incompletely understood.

The phosphatidylinositol 4-phosphate kinase homolog (PIPKH) gene also referred to as PIP5K-like 1 (PIP5KL1) was identified based on sequence similarity to the other PIP5K isoforms^22^. Despite the importance of the PIP5Ks to cellular function, the role of PIPKH is not evident. Initial studies detected the mRNA transcript predominantly in the brain and testis of mice^22^, while more recent proteomic studies have found the protein in gastric epithelial cells^23^. PIPKH was reported to interact with PIP5Kα and β to potentially regulate their activity^22^. Examination of normal and cancerous gastric cancer samples revealed that loss of PIPKH expression occurred in the majority (≈65%) of samples^24^. Additionally, re-expression of PIPKH in the gastric cancer cell line BGC823 resulted in a decrease in both cell migration and proliferation^24^. How PIPKH influences these properties is unclear.

Despite these previous studies, the function of PIPKH within the cell remains unknown. Although bacterially expressed and purified PIPKH does not possess kinase activity, immune-captured epitope-tagged PIPKH from human embryonic kidney 293T cells exhibits low PIP5K activity^22^. However, site-directed mutagenesis of PIPKH suggested that this activity is not intrinsic to the PIPKH^22^. Instead, it was determined that immunoprecipitating PIPKH results in the co-capture of PIP5Kα and PIP5Kβ^22^. Taken together, the prevailing view is that PIPKH is likely a pseudokinase of unknown function. In this study, we aimed to determine the impact of expression of PIPKH in a cell, otherwise lacking this protein.

## Experimental Procedure

### Plasmids

Full-length human PIPKH was synthesized by Integrated DNA Technologies (Coralville, IA, USA) and subcloned into the pIDT-Amp plasmid. PIPKH was amplified by PCR using this plasmid as a template using the following pairs of primers: 5′-CGCGGATCCTCACTCTGTATGGGCTTCTA-3′ and 5′-GCGGTCGACATGGCTGCACCATCACCAGG-3′. The PCR product was introduced into pmCherry-C1 and pEGFP-C1 vector using the restriction enzymes SalI and BamHI site. The GFP/YFP-PIP5Ks were published previously^6^. The following plasmids used in this study have been previously described: GFP-P4Mx2^25^, mCh-p40-PX^26^, GFP-PH-PLCγ and GFP-AKT-PH^27^, GFP-Rab5^28^, GFP-Rab7 and GFP Rab11a^29^, GFP-EEA1^30^, GFP-RILP-C33^31^. The surface charge probes and small G-proteins, GFP-6+, GFP-8+, GFP-K-Ras, and GFP-Rac1, were kind gifts of Dr. Sergio Grinstein (The Hospital for Sick Children, Toronto, ON, CAN)^32^.

### Cell culture and transfection

Chinese hamster ovary (CHO) cells were maintained at 37 °C with 5% CO_2_ in Ham’s F12 and supplemented with 10% fetal bovine serum from Wisent (Burlington, ON, CAN). CHO cells were transiently transfected with plasmids using Fugene 6 (Promega, Madison, WI, USA) according to the manufacturer’s instructions. The following day, 18–24 h post-transfection, cells were observed or fixed with 3.7% paraformaldehyde (Electron Microscopy Sciences, Hatfield, PA, USA) in phosphate-buffered saline for 60 min at room temperature, washed with Tris-buffered saline and stored at 4 °C. Fixed samples were typically imaged within 48 h of fixation.

### Microscopy

#### Confocal microscopy

Fluorescence images were acquired using spinning-disc confocal microscopy or, as indicated, laser scanning microscopy. The spinning-disc confocal system used is located within the Imaging Facility at the Hospital for Sick Children, Toronto, Ontario. This system was assembled by Quorum Technologies (Guelph, Ontario) and is based on an Olympus IX81 with a 60×/1.35 NA oil immersion objective. The system is equipped with diode-pumped solid-state laser lines (440, 491, 561, 638, and 655 nm; Spectral Applied Research), motorized XY stage (Applied Scientific Instrumentation), and a piezo focus drive (Improvision). Images were acquired using a back-thinned, electron-multiplied cooled charge-coupled device (EM-CCD) camera (Hamamatsu Photonics) driven by the Volocity software (version 6.3.0; PerkinElmer).

The laser scanning microscope is located in the St. Michael’s Hospital BioImaging Facility and is a Zeiss LSM 700 inverted confocal microscope with a Plan-Apochromat 60×/1.4 NA oil objective and Zen 2010 software (Zeiss). Analysis of images was performed using Volocity, Zen 2010 or ImageJ software (NIH, Bethesda, MD, USA) depending on the experiment.

#### Fluorescence lifetime measurement-fluorescence resonance energy transfer (FLIM-FRET)

FLIM-FRET measurements were performed at the Hospital for Sick Children Imaging Facility using an Olympus IX81 equipped with light-emitting diodes LED lines (402, 446, 483, and 540 nm) and a Lambert Instruments FLIM (LI-FLIM) attachment. Images were acquired using a 150x/1.45 NA oil immersion objective and a Hamamatsu C9100-13 Electron Multiplying (EM)-CCD camera using the 483 and 540 LED excitation lines. The LI-FLIM v1.2.12 (Lambert Instruments, Groningen, Netherlands, NLD) was used to monitor the fluorescence lifetimes of the donor fluorophores.

### Post-acquisition analysis

The degree of colocalization was determined by Pearson’s Correlation Coefficient (PCC), or simply “Pearson’s”, using the JACoP plug-in in ImageJ. Specifically, cells were selected using the freehand tool in ImageJ. Images were then split into the two contributing channels for further analysis. For consistent and reproducible analysis, auto-thresholding for the two channels was performed using the Yen algorithm, as previously described, followed by the determination of the Pearson’s^33^.

For determining the relative distribution of fluorescent probes or proteins in the cell, the ROI tool was used to highlight areas or the plasma membrane, cytoplasm or background (outside of the cell) and determine the mean intensity per pixel. The percent of the plasmalemmal resident probe was determined by subtracting the integrated cytoplasmic signal from the total cellular fluorescence and represented as a percent of the total cellular fluorescence.

## Results

### PIPKH recruits PIP5Ks to intracellular compartments and interacts with PIP5Ks in cells

**Figure 1:**
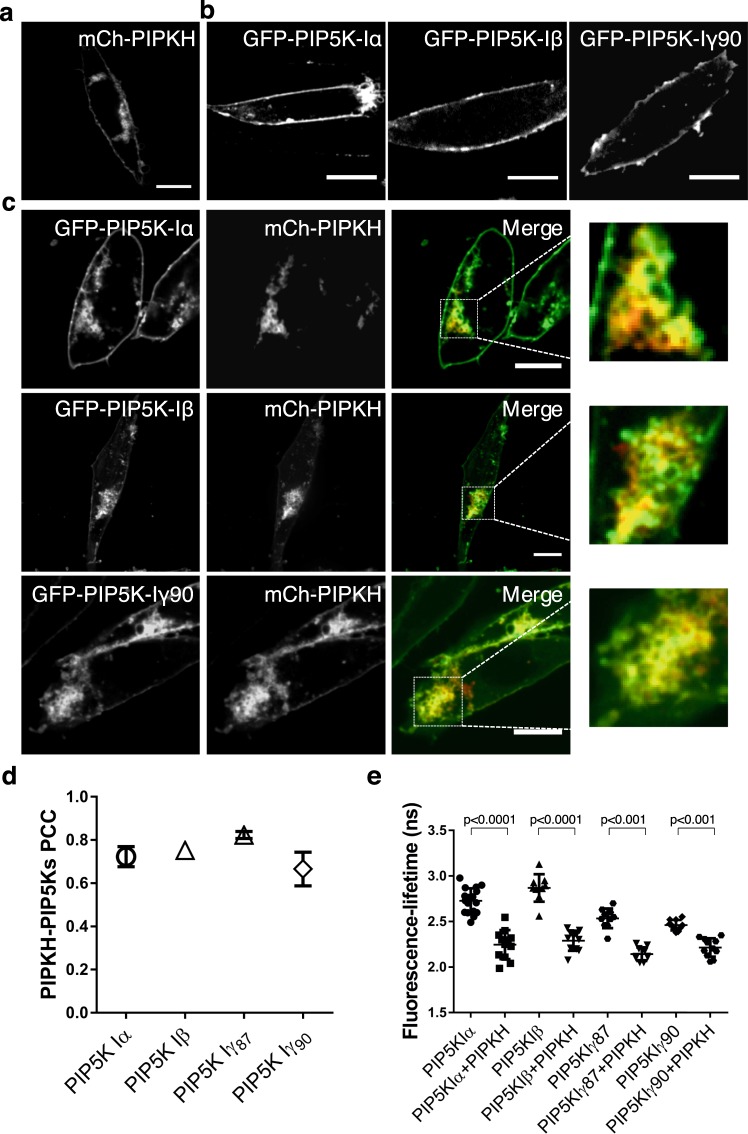
Overexpression of PIPKH redistributes PIP5Ks. (**a**) PIPKH localizes to the plasma membrane (PM) and endomembranes. Chinese Hamster Ovary (CHO) cells were transiently transfected with mCherry (mCh)-tagged PIPKH and observed by spinning disc confocal microscopy. (**b**) Type I phosphatidylinositol 4-phosphate 5-kinases (PIP5Ks) localize predominantly to the PM in CHO cells. Cells transiently transfected with GFP or YFP chimeras of PIP5K isoforms. (**c**) PIPKH overexpression causes a redistribution of PIP5Ks to internal structures. CHO cells cotransfected with the indicated YFP- or GFP-tagged PIP5K isoforms and mCh-tagged PIPKH. Images were acquired using spinning-disc confocal microscopy. Scale bars = 10 μm. (**d**) PIPKH and PIP5Ks extensively colocalize. Pearson’s correlation coefficient (PCC) was determined for each of the indicated PIP5K isoforms and PIPKH. Values are the mean ± std. dev. from a minimum of 5 cells per experiment imaged from at least three different experiments (n = 3). (**e**) Fluorescently tagged versions of PIPKH and PIP5Ks are in close proximity to each other. Fluorescence Lifetime Imaging Microscopy-Forster Resonance Energy Transfer (FLIM-FRET) was used to examine the proximity of the GFP or YFP (donor) to the mC-PIPKH (acceptor). Values present the mean lifetimes ± std. dev. from a minimum of 9 cells observed in three separate experiments (n = 3). Significance testing was performed using unpaired two-tailed Student’s *t-test* with Welch correction.

We first examined the subcellular distribution of heterologously expressed human PIPKH in Chinese hamster ovary (CHO) cells. CHO cells transiently transfected with plasmids encoding mCherry (mCh)-tagged PIPKH were examined using spinning-disc confocal microscopy. As shown in Fig. 1a and Sup. Fig. 1, mCh-PIPKH was expressed as an intact and stable chimeric protein that localized to the plasma membrane as well as cytoplasmic membranes. A previous study demonstrated that recombinant PIPKH does not possess the ability to bind liposomes *in vitro*^22^. Thus, we suspected that the localization of the PIPKH is a result of protein-protein interactions. To date, the only known interacting partners for PIPKH are PIP5Kα and PIP5Kβ. Previously, we had demonstrated that the Type I PIP5Ks localize primarily to the plasma membrane in a macrophage cell line, and we confirmed this observation using GFP or YFP-chimeras in CHO cells (Fig. 1b)^6^. This raises the possibility that the plasmalemmal pool of PIPKH was due to interactions with the α and β isoforms. When co-expressed, we found that PIPKH caused relocalization of not only PIP5K α and β but also the γ isoform to internal membranes (Fig. 1c,d). Thus, enforced expression of PIPKH was able to override the endogenous targeting determinants of the PIP5Ks.

To determine if the PIPKH induced relocalization of the PIP5Ks was due to protein-protein interactions, we used the fluorescence-resonance-energy transfer (FRET) to detect the proximity of GFP/YFP-tagged PIP5K and mCh-PIPKH. More specifically, we took advantage of fluorescence lifetime imaging microscopy (FLIM) or so-called “FLIM-FRET” that obviates many of the technical issues associated with traditional FRET. This method of determining FRET is based on a decrease in the fluorescence lifetime, the time in nanoseconds between excitation of the donor fluorophore and the emission of light, when proximal to a suitable acceptor fluorophore. Thus, FLIM depends on the molecular environment of a fluorophore and not its concentration^34^. Intensity-based FRET methods relies on the FRET efficiency calculated from the ratio of emission intensities from donor and acceptor in the absence and presence of energy transfer. These measurements can be quite susceptible to “bleed-through” of donor and acceptor fluorophores and variations in the expression level of the partner proteins^34^. FLIM-FRET also minimizes the photobleaching of the acceptor fluorophore a confounding issue that can result in decreased apparent FRET efficiency^35^. Additionally, since only the donor is excited during FLIM-FRET, it is less dependent on local concentration variations and fluctuations observed in live cells^35^. Finally, FLIM-FRET can use a broader range of fluorophores and dark acceptors than FRET^36^. Using FLIM-FRET, a decreased fluorescence lifetime of YFP-PIP5Kα/β and GFP-PIP5Kγ87/γ90 in cells that were transfected with mCh-PIPKH was observed (Fig. 1e). This result is consistent with the previous finding that demonstrated PIPKH and the α and β isoforms could co-immunoprecipitate, supporting the notion that they directly interact or could be part of a larger complex^22^. These results suggest that PIPKH can heterodimerize with PIP5K isoforms and alter the targeting of the kinases.

### PIPKH causes intracellular accumulation of PtdIns(4,5)P_2_

**Figure 2:**
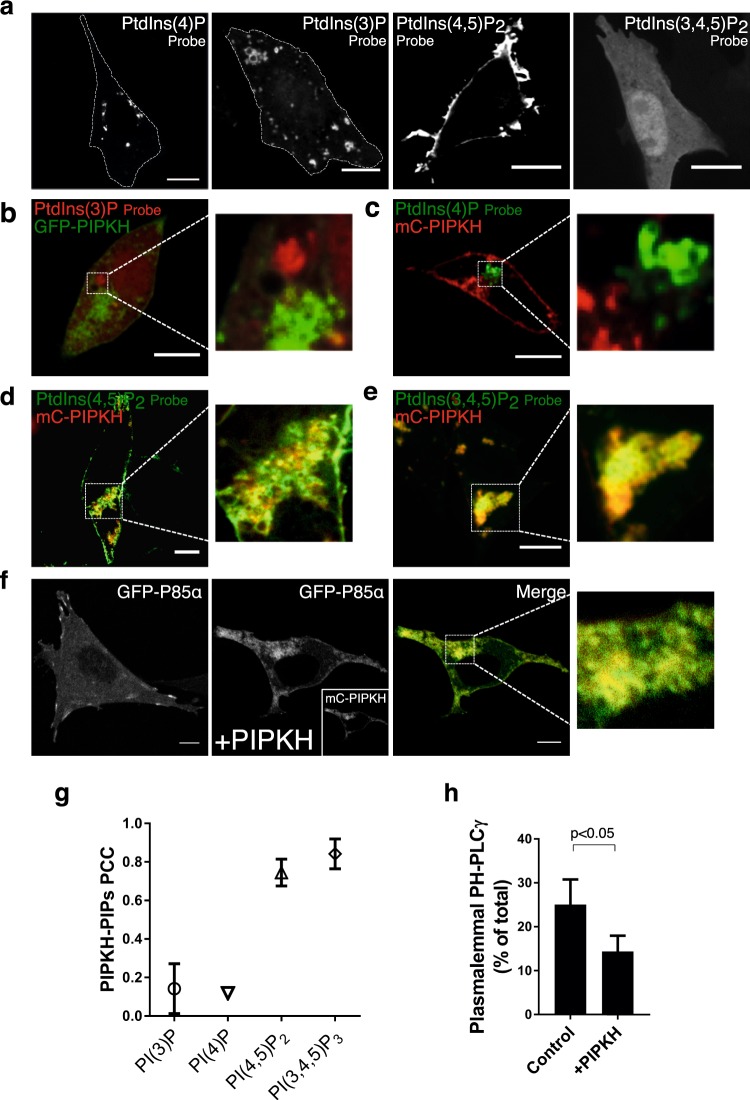
Expression of PIPKH results in an aberrant distribution of PtdIns(4,5)P_2_ and PtdIns(3,4,5)P_3_. (**a**) Distribution of phosphoinositides in CHO cells. CHO cells were transiently transfected with fluorescently-tagged probes for PtdIns(3)P (PX), PtdIns(4)P (tandem P4M), PtdIns(4,5)P_2_ (PH-PLCδ), and PtdIns(3,4)P_2_/PtdIns(3,4,5)P_3_ (PH-AKT). (**b**,**c**) PIPKH does not co-localize with monophosphorylated phosphatidylinositol. CHO cells were transiently co-transfected with mC-PX and GFP-PIPKH or GFP-2xP4M and mC-PIPKH were examined 18–24 h post-transfection. While both phosphoinositide probes decorate cytoplasmic structures, these are devoid of PIPKH. (**d**,**e**) PIPKH causes altered polyphosphoinositide distribution. CHO cells transiently co-transfected with mC-PIPKH and either GFP-PH-PLCδ or PH-AKT were examined 18 h post-transfection. PIPKH overlaps extensively with cytoplasmic pools of PtdIns(4,5)P_2_ and PtdIns(3,4)P_2_/PtdIns(3,4,5)P_3_. Scale bars = 10 μm. (**f**) CHO cells transiently expressing PIPKH and the p85α subunit of phosphatidylinositol 3-kinase (PI3K) displayed relocalization of the p85α from the plasma membrane to endosomes. Scale bars = 10 μm. (**g**) Poly-phosphoinositides colocalize with PIPKH. Pearson’s correlation coefficient of PIPKH with each of the indicated phosphoinositide probes. Values represent the mean ± std. dev. from a minimum of 5 cells per experiment imaged from at least three different experiments (n = 3). (**h**) Enforced expression alters the relative distribution of PtdIns(4,5)P_2_ in the cell. A histogram of the fraction of the total cellular PH-PLCδ probe in proximity to the plasma membrane. The data represent the mean ± std. dev. from a minimum of 5 cells per experiment from at least three different experiments (n = 3). Significance testing was performed using unpaired two-tailed Student’s *t-test* with Welch correction.

A prevailing theory is that the presence of individual phosphoinositide species serves as molecular beacons to recruit peripheral proteins and provide identity to the specific organelles. As expression of PIPKH caused the marked redistribution of PIP5Ks to endomembranes, we wanted to determine if PIPKH could also alter the distribution of PIPs. First, we used several commonly-used genetically encoded biosensors to monitor the distribution and relative abundance of individual PIP species. As depicted in Fig. 2a, CHO cells have intracellular pools of PtdIns(4)P (as monitored by GFP-P4M), PtdIns(3)P (mCh-PX) and PtdIns(4,5)P_2_ (PH-PLCδ). However, these cells have minimal PtdIns(3,4)P_2_ or PtdIns(3,4,5)P_3_ as monitored by PH-AKT and BTK-PH (Fig. 2a and Sup. Fig. 2). Next, we cotransfected these probes together with the PIPKH plasmid and imaged using confocal microscopy. As depicted in Fig. 2b,c,g, PIPKH displayed minimal overlap with monophosphorylated PIPs. However, mCh-PIPKH caused a marked increase in both PtdIns(4,5)P_2_ and PtdIns(3,4)P_2_/PtdIns(3,4,5)P_3_ on the endomembranes (Fig. 2d,e) and, similar to the localization of the PIP5Ks, the p85α subunit of the class I PI3K was also relocalized (Fig. 2f). Additionally, the intracellular pools of PIPs and p85α displayed robust colocalization with PIPKH (Fig. 2g). Previous results in human embryonic kidney 293 cells had demonstrated that PIPKH does not impact the total cellular levels of PtdIns(4,5)P_2_^22^. Here, we determined the relative distribution of the PtdIns(4,5)P_2_ probe in the plasma membrane vs. the rest of the cell. In control cells, ≈25% of the mCh-PH-PLCδ is associated with the plasma membrane, while in cells expressing PIPKH, only ≈15% of the PtdIns(4,5)P_2_ probe is now found in the plasma membrane (Fig. 2h).

### PIPKH interacts with PIP5Kγ87 independent of PtdIns(4,5)P_2_

**Figure 3:**
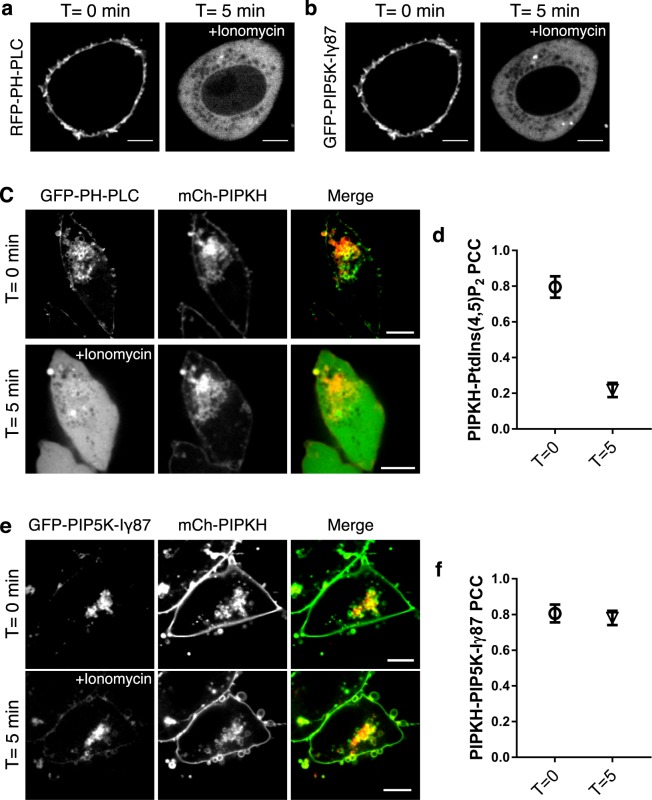
PIPKH binds to endosomes and interacts with PIP5Kγ87 independent of PtdIns(4,5)P_2_. (**a**,**b**) PH-PLCδ and PIP5Kγ87 localize primarily to the plasma membrane in resting conditions but are rapidly released to the cytosol following the addition of the calcium ionophore ionomycin. Chinese hamster ovary (CHO) cells were transiently transfected with GFP-PH-PLCδ or GFP-PIP5Kγ87, treated with 10 μM ionomycin and observed by confocal microscopy at T = 0 and T = 5 min. (**c**) The endosomal pool of PtdIns(4,5)P_2_ but not PIPKH is depleted in cells within 5 min of adding ionomycin. CHO cells were transiently co-transfected with mCherry (mC)-PIPKH and GFP-PH-PLCδ, treated with 10 μM ionomycin and observed by confocal microscopy at T = 0 and T = 5 min. (**d**) Pearson’s correlation coefficient of PIPKH and PH-PLCδ at the indicated times of ionomycin addition. Values represent the mean ± std. dev. from a minimum of 5 cells per experiment, imaged from at least three different experiments (n = 3). (**e**) Depletion of endosomal PtdIns(4,5)P_2_ does not displace PIP5Kγ87 from endosomes. CHO cells were transiently co-transfected with mC-PIPKH and GFP-PIP5Kγ87 were treated with 10 μM ionomycin and observed by confocal microscopy at T = 0 and T = 5 min. (**f**) PIP5Kγ87 colocalizes with PIPKH in the absence of PtdIns(4,5)P_2_. Pearson’s correlation coefficient of PIPKH with PIP5Kγ87 at different time points of post ionomycin treatment. Values represent the mean ± std. dev. from a minimum of 5 cells per experiment imaged from at least three different experiments (n = 3). Significance testing was performed using unpaired two-tailed Student’s *t-test* with Welch correction. Scale bars = 10 μm.

PIP5Ks target to the plasma membrane in part due to negative surface charge, while rapid depletion of PtdIns(4,5)P_2_ results in their displacement^6^. Since PIPKH causes the redistribution of PIP5Ks to endosomes and the appearance of PtdIns(4,5)P_2_ on these structures we next considered if PtdIns(4,5)P_2_ was required to support the interactions between PIPKH and PIP5K. The increase of cytosolic calcium by the addition of ionomycin is known to activate phospholipase Cγ and degrade PtdIns(4,5)P_2_^37^. Consistent with previously published results, we find that both the PH-PLCδ and PIP5Kγ87 are displaced from the plasma membrane within 5 min of ionomycin addition (Fig. 3a,b). In cells expressing PIPKH, the large endosomal pool of PtdIns(4,5)P_2_ is also depleted within 5 min of addition of the calcium ionophore (Fig. 3c). However, loss of PtdIns(4,5)P_2_ from the endocytic compartments has minimal impact on the localization of the PIPKH (Fig. 3c,d). In parallel experiments using the same conditions, we find that the PIP5Kγ87 remains colocalized with PIPKH on endocytic structures despite the sharp increase in cytosolic calcium (Fig. 3e,f). Together these results suggest that PIP5Kγ87 and possible other PIP5Ks interact with PIPKH independent of the strong negative surface charge of the membrane.

### PIPKH localizes to multiple endocytic compartments

**Figure 4:**
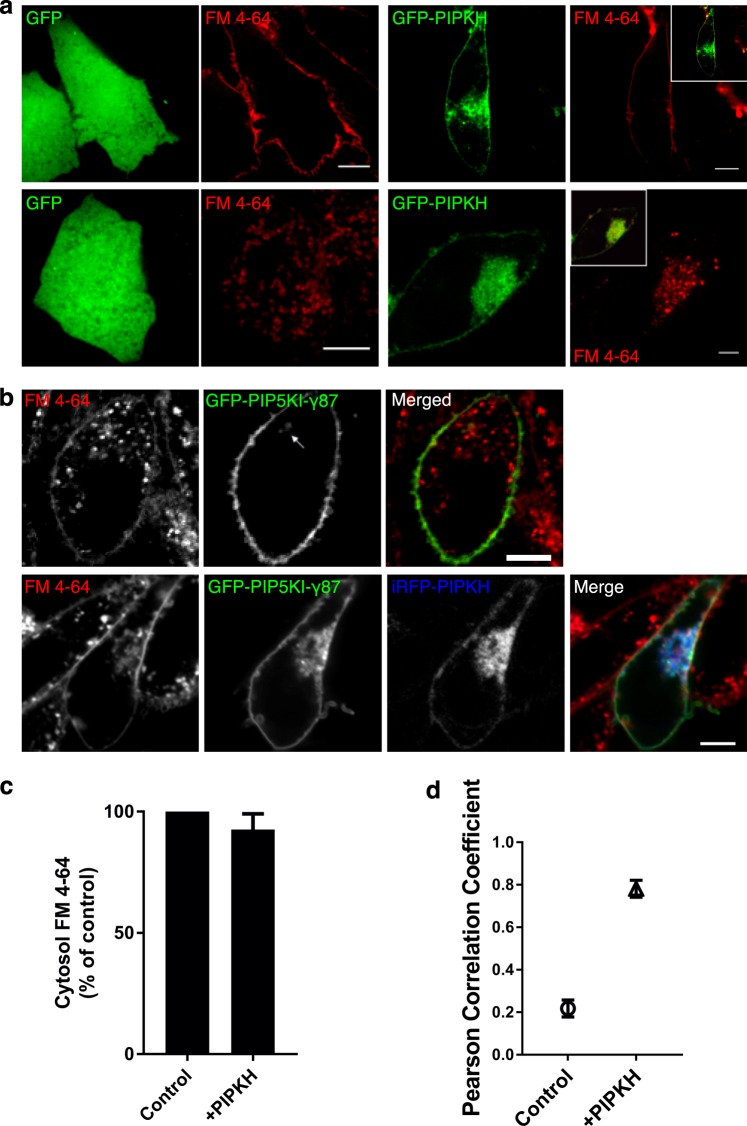
The PIPKH-positive compartment is endocytic and detached from the plasma membrane. (**a**) Cells expressing GFP or GFP-PIPKH were stained with FM4-64 for 30 min at either 4 °C or 37 °C, washed with PBS and observed by confocal microscopy. Scale bars = 10 μm. (**b**) FM4-64 labeling of cells expressing GFP-PIP5Kγ87 or co-expressing infrared fluorescent protein (iRFP)-PIPKH and GFP-PIP5Kγ87. Cells were stained with FM4-64 for 30 min at 37 °C and observed by confocal microscopy. The arrow highlights an endocytic structure that contains a portion of the GFP signal. Scale bars = 10 μm. (**c**) Most FM4-64 positive internal structures (endosomes), have little PIP5Kγ87 associated in control cells. However, PIPKH co-expression localizes PIP5Kg87 to endocytic membranes. Pearson’s correlation coefficient (PCC) of PIP5Kγ87 with FM4-64. Values represent the mean ± std. dev. from a minimum of 5 cells per experiment imaged from at least three different experiments (n = 3). (**d**) The relative endocytosis of FM4-64 was minimally impacted by expression of the PIPKH protein. A histogram of the relative abundance of FM4-64 in control and PIPKH-overexpressing cells. The data represent the mean ± std. dev. from a minimum of 5 cells per experiment from at least three different experiments (n = 3).

We suspected that the cytoplasmic PIPKH resides on endosomes. However, since alterations in PtdIns(4,5)P_2_ can prevent the scission of endocytic carriers, we wanted to make sure that the PIPKH-positive membranes were not connected to the plasma membrane and were indeed endocytic in nature^38–40^. To address both of these issues, we stained cells expressing GFP-PIPKH with the membrane dye FM4-64 at 4 °C to label the plasma membrane and at 37 °C to label the endocytic compartments. Cells expressing GFP, GFP-PIPKH or untransfected cells in the field of view all displayed plasmalemmal staining at 4 °C (Fig. 4a). Since FM4-64 can diffuse rapidly in the plane of the membrane, this demonstrates that the PIPKH structures are not invaginations or tubules that remain connected to the plasma membrane. Next, we incubated cells growing at 37 °C with a 30 min continuous pulse of FM4-64 to label endocytic compartments. To improve the visualization of the endosomes, we washed the cells three times with PBS to remove plasmalemmal FM4-64 before imaging. Both GFP and GFP-PIPKH expressing cells have numerous FM4-64 positive endosomes (Fig. 4a, bottom panel). Similar to the PIP5Ks, we found that the FM4-64 also displayed a clustered phenotype in GFP-PIPKH-expression cells.

Typically, the majority of PIP5K signal is associated with the plasma membrane, but occasionally small FM4-64 positive structures can be observed (Fig. 4b, arrow). However, FM4-64 labeling of cells co-expressing infrared fluorescent protein (iRFP)-PIPKH and GFP-PIP5K revealed extensive colocalization of the PIP5K and FM4-64 signals (Fig. 4b,c) confirming that the GFP-PIP5K is relocalized to clustered endocytic compartments. Despite the clustered nature of the endocytic compartment, we find that the relative endocytosis of FM4-64 was minimally impacted by the expression of the PIPKH protein (Fig. 4d).

**Figure 5:**
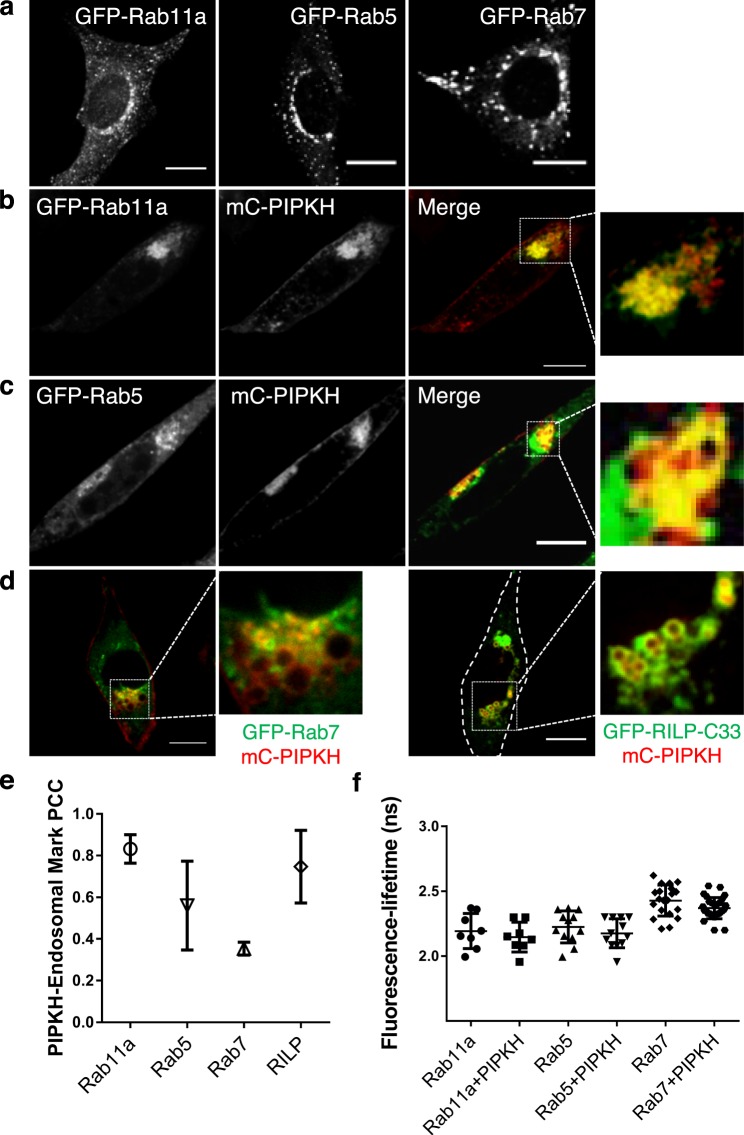
PIPKH colocalizes with endocytic Rab proteins. (**a**) Distribution of endocytic Rab proteins in Chinese hamster ovary (CHO) cells. CHO cells were transiently transfected with GFP tagged versions of Rab11a, Rab5, and Rab7 and imaged by confocal microscopy 18 h post-transfection. (**b**,**c**) PIPKH colocalizes with Rab11a and Rab5 on clustered endosomes. CHO cells were cotransfected with mCherry (mC)-PIPKH and GFP chimeras of either Rab11a or Rab5 and images captured using spinning disc confocal microscopy. (**d**) PIPKH localizes with activated Rab7. CHO cells were co-transfected with mC-PIPKH and GFP chimeras of either Rab7 or C-terminal half of Rab7-interacting lysosomal protein (RILP-C33) and imaged by spinning disc confocal microscopy. (**e**) Pearson’s correlation coefficient for the indicated Rab proteins, active Rab7 and PIPKH. Values represent the mean ± std. dev. from a minimum of 5 cells per experiment from at least three different experiments (n = 3). (**f**) PIPKH colocalizes with but is not in proximity to the indicated Rab proteins. Distribution of fluorescence lifetimes of GFP (donor) tagged to the specific Rab protein and mC-PIPKH. Values are the mean ± std. dev. from a minimum of 15 technical replicates from at least three different experiments. Statistical testing was performed using unpaired *t-test* with Welch correction. Note: there is no significant difference in lifetime between donor alone and donor plus acceptor for these pairs of proteins. Scale bars = 10 μm.

Next, we used GFP-chimeras of Rab proteins to identify specific endosomal compartments: Rab11a (recycling endosomes), Rab5 (early endosomes) and Rab7 (late endosomes/lysosomes). In control CHO cells, the Rab11a-positive recycling endosomes tend to cluster around the nucleus and near the microtubule organizing center, while the Rab5 and Rab7-positive populations have a more dispersed appearance (Fig. 5a). As illustrated in Fig. 5b–d, many of the PIPKH-induced clustered endosomes co-localized with Rab11a, Rab5, or Rab7 with the strongest colocalization occurs with Rab11a and Rab5 (Fig. 5b,e). We wondered whether our colocalization analysis for Rab7 appeared artificially low since the transient transfection of GFP-Rab7 repeatedly gave us high cytosolic signals. To circumvent this concern, we compared the localization of the PIPKH with the Rab7-interacting lysosomal protein (RILP) an effector that specifically binds to the active form of Rab7 (GTP-Rab7) (Fig. 5d)^31^. When colocalization experiments were conducted with RILP-C33 (C-terminal half of RILP that is capable of binding to GTP-Rab7) and PIPKH, we found that RILP-C33 also showed robust colocalization with PIPKH similar to Rab11a and Rab5. Additionally, this experiment revealed that many of the clustered endosomes still have active Rab7. Considering the strong colocalization with several Rab proteins, we next considered whether PIPKH could also be binding to Rab proteins. To investigate this possibility, we again conducted FLIM-FRET analysis and determined that the possibility of PIPKH interacting with Rab proteins is unlikely (Fig. 5f). Collectively, these results suggest that PIPKH can localize to multiple types of endosomes and further influences their distribution.

**Figure 6:**
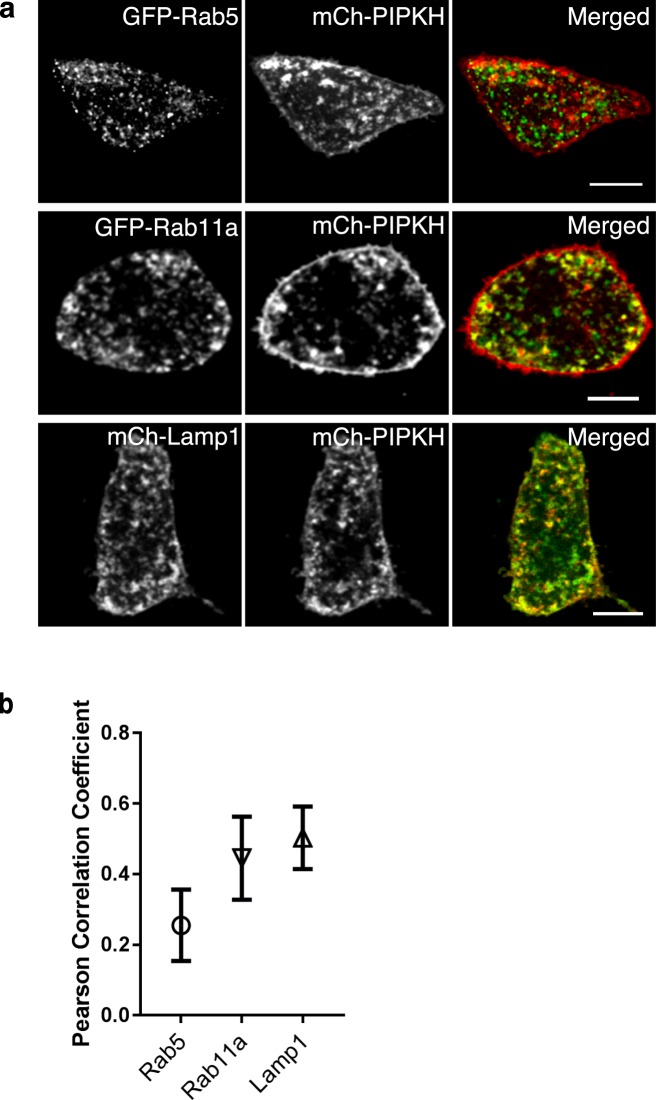
Microtubules are required for PIPKH-induced endosomal clustering. (**a**) Chinese hamster ovary cells were transiently transfected with GFP tagged versions of Rab11a, Rab5, or Lamp1 and mCherry-PIPKH, and treated with colchicine to prevent the formation of microtubules and grown for 18 hours followed by live cell imaging. Co-localization of PIPKH with Rab11a and Lamp1, a marker of late endosomes and lysosomes was observed, whereas Rab5 showed less co-localization. Scale bars = 10 μm. (**b**) Pearson’s correlation coefficient for the indicated Rab proteins, Lamp1 and PIPKH. Values represent the mean ± std. dev. from a minimum of 9 cells per experiment from at least three different experiments (n = 3).

From the previous experiment and the clustering of the endosomes, it was difficult to determine if PIPKH had a preference for any individual type of endosome or whether the endosomes had become “hybrid” compartments containing markers of early, late and recycling endosomes. Endosomes are known to move throughout the cell by the action of microtubule-based motors. Thus, we re-examined the localization of PIPKH with the endosomal markers in cells with depolymerized microtubules. In this experiment, cells were transfected with the indicated plasmids and treated with colchicine to prevent the formation of microtubules and grown for 18 hours, followed by live-cell imaging. Interestingly, the mCh-PIPKH still labels the endosomes and plasma membrane but now the endocytic compartments are dispersed throughout the cell (Fig. 6a). In this experiment, comparable co-localization was detected for Rab11a and Lamp1, a marker of LEs and lysosomes, whereas Rab5 showed minimal co-localization. Currently, it is unclear why PIPKH targets only a subset of cytoplasmic membranes and endosomes.

### PIPKH causes the relocalization of polybasic plasmalemmal proteins K-Ras and Rac1

**Figure 7:**
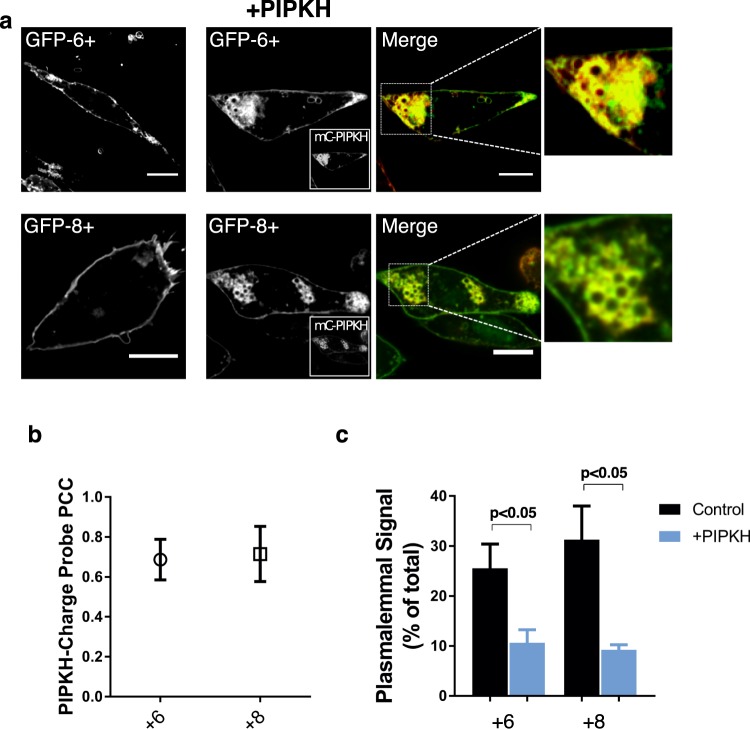
Enhanced expression of PIPKH alters the membrane surface charge of endosomal compartments. (**a**) Expression of PIPKH causes a redistribution of surface charge sensitive probes. Chinese hamster ovary (CHO) cells were transiently transfected with GFP tagged charge probes, +6 and +8, or were transiently co-transfected with mCherry (mC)-PIPKH and GFP-+6 or GFP-+8 charge probe. Images were captured using spinning-disc confocal microscopy 18 h post-transfection. (**b**) PIPKH colocalizes with the negative surface charge detectors. Pearson’s correlation coefficient plot for PIPKH and GFP-+6 and GFP-+8 is depicted. Values are the mean ± std. dev. from a minimum of 5 technical replicates from at least three different experiments (n = 3). (**c**) PIPKH stimulated alterations in phosphoinositide distribution cause a decrease in the amount of charge probe associated with the plasma membrane (PM). The histogram represents the percent of charge probe associated with the PM in control and PIPKH-expressing cells. The percentages ± std. dev. from a minimum of 15 cells from three different experiments. Statistical testing was performed using a paired two-tailed Student’s *t-test* with Welch correction.

**Figure 8:**
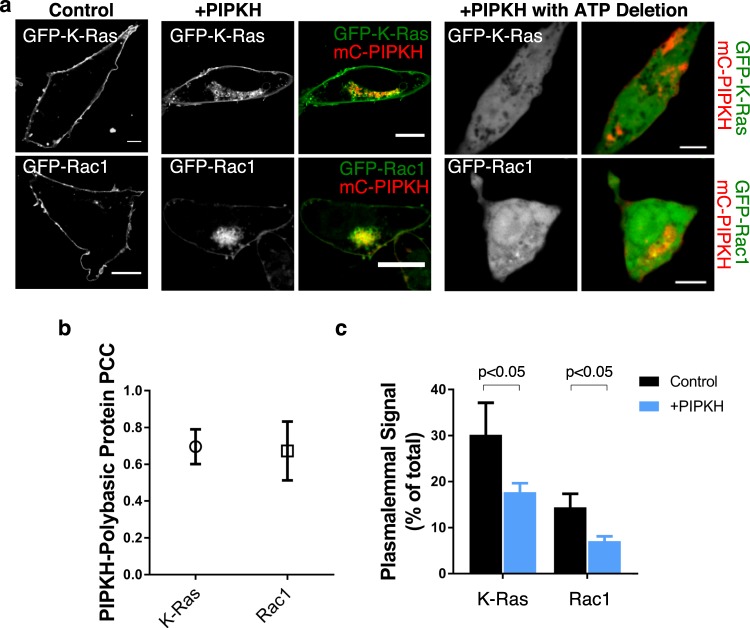
PIPKH overexpression relocalizes K-Ras and Rac1 to endosomal compartments. (**a**) Expression of PIPKH causes a redistribution of small G-proteins with polybasic tails. Chinese hamster ovary cells were transiently transfected with GFP tagged K-Ras and Rac1 or were transiently co-transfected with mCherry(mC)-PIPKH and GFP-Rac1 or GFP-K-Ras. Images were captured using spinning-disc confocal microscopy 18 h post-transfection. To deplete cellular level ATP levels cell were incubated with 2-deoxyglucose and antimycin. (**b**) PIPKH causes accumulation of the small G-proteins on endosomes. Pearson’s correlation coefficient for mC-PIPKH and GFP-K-Ras and GFP-Rac1 are plotted. Values are the mean ± std. dev. from a minimum of 5 technical replicates from at least three different experiments (n = 3). (**c**) PIPKH stimulated alterations in phosphoinositide distribution cause a decrease in the amount of K-Ras and Rac1 associated with the plasma membrane (PM). The histogram represents the percent of the indicated small G-protein associated with the PM in control and PIPKH-expressing cells. The percentages ± std. dev. from a minimum of 5 technical replicates per biological replicate with n = 3. Statistical testing was performed using a paired two-tailed Student’s *t-test* with Welch correction.

We hypothesized that PIPKH-induced aberrant PtdIns(4,5)P_2_ distribution would cause an increase in the negative charge density on endosomal compartments. To test this hypothesis, CHO cells were transfected or cotransfected with surface charge biosensors and PIPKH. CHO cells transfected with the surface charge biosensors alone, +8 and +6 probes, localized preferentially to the plasma membranes consistent with previous findings (Fig. 7a–c)^32^. In contrast, in CHO cells transiently expressing PIPKH, the surface charge biosensors co-localized with PIPKH to endosomal compartments, suggesting that significant phosphoinositide is being generated to impact the charge probes. Next, we extended this finding to two small GTPases that use polycationic patches to localize to the plasma membrane, K-Ras and Rac1. To do this, we transiently co-transfected K-Ras or Rac1 with PIPKH in CHO cells. Compared to control cells, both K-Ras and Rac-1 were observed to relocalize from the plasma membrane to PIPKH-positive endosomal compartments (Fig. 8a–c). The impact of PIPKH on K-Ras and Rac1 relied on the presence of phosphoinositides as depletion of ATP a maneuver known to abolish phosphoinositides resulted in the two G-proteins residing in the cytosol. These results indicate that PIPKH-induced alterations in the cellular distribution of PIPs are sufficient to cause relocalization of proteins that generally target to the plasma membrane via electrostatic interactions. These results support the observation that the degree of negative surface charge on a membrane can dictate the association of peripheral membrane proteins.

## Discussion

Here, we have assessed the ability of the pseudokinase PIPKH to influence the subcellular distribution of the PIP5Ks and PtdIns(4,5)P_2_ within the cell. Cells overexpressing mCh-PIPKH showed different localization patterns for the four PIP5Ks examined, consistent with a previous report that the impact of PIPKH on PIP5Ks appears to be direct protein-protein interactions^22^. We found that the fluorescence lifetime of the GFP/YFP-tagged versions of PIP5Ks was decreased in the presence of mCh-PIPKH. Together these results suggest that PIPKH can interact with PIP5Ks and thereby regulate their subcellular distribution. Intriguingly, the relocalization of PIP5Ks and/or the accumulation of endosomal PtdIns(4,5)P_2_ was associated with clustering of endosomes in a perinuclear region. However, the mechanism driving this apparent clustering is unclear. One possibility is that peripheral proteins associated with the limiting membrane of the endosomes may be altered following PIPKH expression. Indeed, the polyvalent anionic PIPs confer a negative surface charge on the inner leaflet of the plasma membrane, which is critical for the targeting of many plasmalemmal proteins^7,8^. In support of this notion, we find that changes in cellular PtdIns(4,5)P_2_ distribution cause mislocalization of plasmalemmal proteins to PIPKH-/PtdIns(4,5)P_2_-positive endosomes. Our results suggest that the endogenous function of PIPKH may include acting as a regulator for one or more of the PIP5Ks to modulate the abundance of PtdIns(4,5)P_2_ in the endocytic pathway.

The regulation of PIP5K activity and cellular localization is essential for generating specific cellular pools or local concentrations of PtdIns(4,5)P_2_. It is well known that a 25-amino acid segment, named the activation loop, confers PIP5Ks substrate specificity to PtdIns(4)P^15^. PIP5Ks colocalize with their product PtdIns(4,5)P_2_, in the plasma membrane in a variety of cell types, including macrophages, by virtue of a positively charged face^6^. Several proteins influence the activity of PIP5K isoforms via direct interactions^41–43^. These include small GTPases Rho, Rac and ARF that have been described to aid in the targeting of PIP5Ks *in vivo* and stimulate the kinase activity *in vitro*^19–21,43^. These interactions may help facilitate the generation of specific pools of PtdIns(4,5)P_2_. For instance, Rac has been demonstrated to influence PIP5Kβ at the plasma membrane during neurite retraction^44^. The PH-PLCδ reporter primarily localizes to the plasma membrane, yet PIP5Ks and their product have also been observed in a variety of other, more challenging to observe, subcellular locations, including endosomes, autophagolysosomes and the nucleus^45–50^. We suspect that many of these specific pools will be controlled by a variety of factors, including membrane surface charge, substrate availability and protein-protein interactions.

Unfortunately, the endogenous function of PIPKH remains unclear in mammals. Proteomics and expression data suggest that PIPKH is present in gastric epithelial cells^23^ and the lens of the eye in mice. PIPKH is lost in a subset of gastric cancer samples that correlated with enhanced cell proliferation and migration^24^. Notable, Shi *et al*. reported that expression of PIPKH in the human gastric cancer line BGC-823 inhibited the phosphorylation of AKT at Ser^473^, but the mechanism is unknown^24^. Akt is recruited to the membrane by interaction with PtdIns(3,4,5)P_3_ and subsequently phosphorylated by phosphoinositide-dependent kinase (PDK) on Thr^308^ and by mTOR on Ser^473^ to become fully activated^51,52^. Our results suggest that PIPKH-induced alterations in the cellular distribution of PIPs may negatively affect the recruitment of AKT to the plasma membrane. Other possibilities are that AKT is recruited to PtdIns(3,4,5)P_3_ containing endosomes, but mTOR is not present or that expression of PIPKH activates PHLPP isoforms (PH domain and Leucine-rich repeat Protein Phosphatases) that dephosphorylates AKT. Further work towards improving understanding of the biophysical properties of PIPKH is required for revealing its precise role(s) in cancer metastasis.

Gastric epithelial cells express caveolin and possess caveolae. In this regard, the only other study we are aware of that has directly investigated the cellular impact of PIPKH was a siRNA-based screen of the human kinome to identify regulators of caveolae^15^. In this study, Pelkmans and Zerial discovered that silencing of PIPKH in HeLa cells led to an increase of multi-caveolar assemblies or “rosettes” at the expense of individual caveola^53^. Mechanistically it is unclear how or why this occurred. The main caveat of this study was that the efficiency and specificity of the silencing were not investigated. To date, it remains unclear what signals control the formation of higher-order caveolar assemblies compared to the traditional stand-alone caveola.

In summary, our results indicate that PIPKH may function as a regulatory protein for PIP5Ks. Based on its localization, we suspect that PIPKH supports the production of a specialized pool of PtdIns(4,5)P_2_ in the endocytic pathway. Collectively, our results, together with previous work, suggest that PIPKH may be necessary only in a specialized cell type such as gastric epithelial cells. However, due to its preservation over a long evolutionary time scale, an alternative possibility is that it supports a key developmental process not present in immortalized cultured cells. This will require further investigation in the future.

## Supplementary information

Supplementary information
