# Supplementary material for: Enforced expression of phosphatidylinositol 4-phosphate 5-kinase homolog alters PtdIns(4,5)P2 distribution and the localization of small G-proteins
Source: Sci Rep. 2019 Oct 15;9:14789. doi: 10.1038/s41598-019-51272-z (PMC6794296; doi:10.1038/s41598-019-51272-z)
Supplement: Supplementary file 1 — Supplementary information [file 41598_2019_51272_MOESM1_ESM.pdf]

Enforced expression of phosphatidylinositol 4-phosphate 5-kinase homolog alters PtdIns(4,5)P_2 distribution and the localization of small G-proteins

Yanbo Yang, Miriam Park, Masashi Maekawa and Gregory D. Fairn

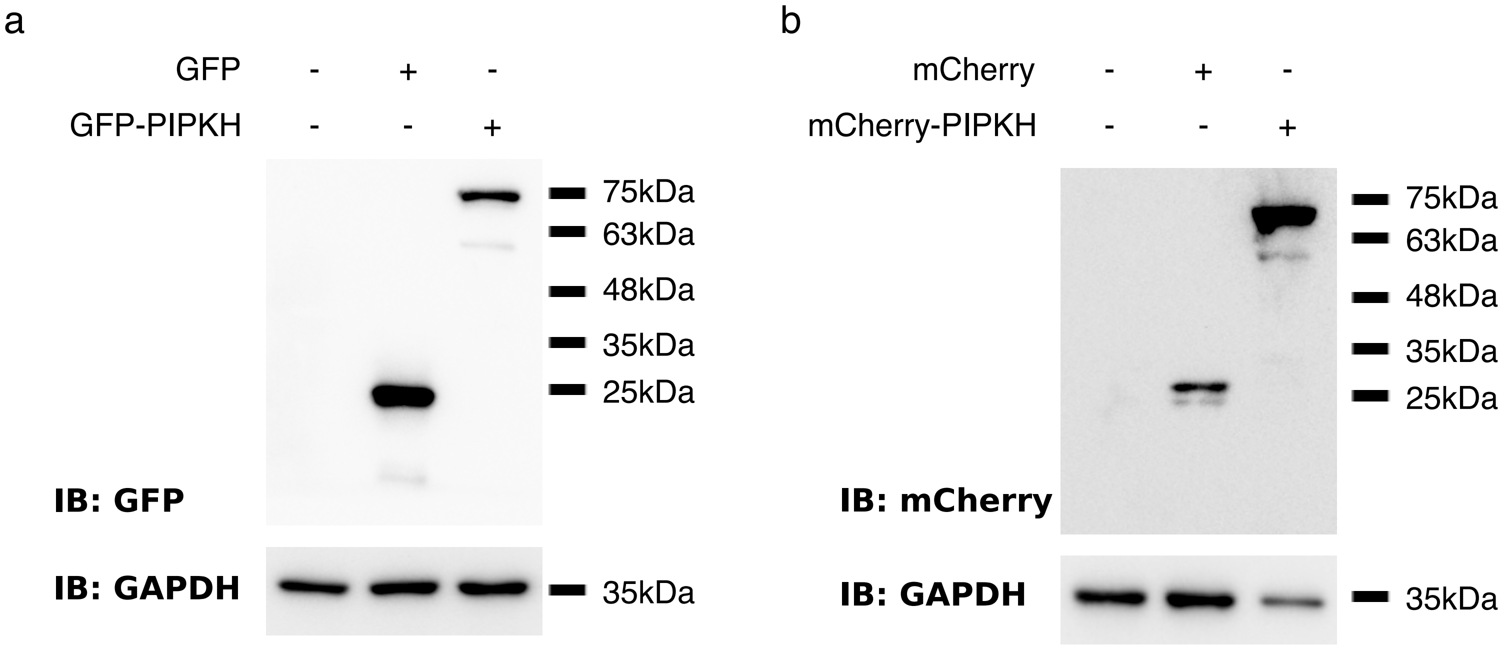

Source Blots

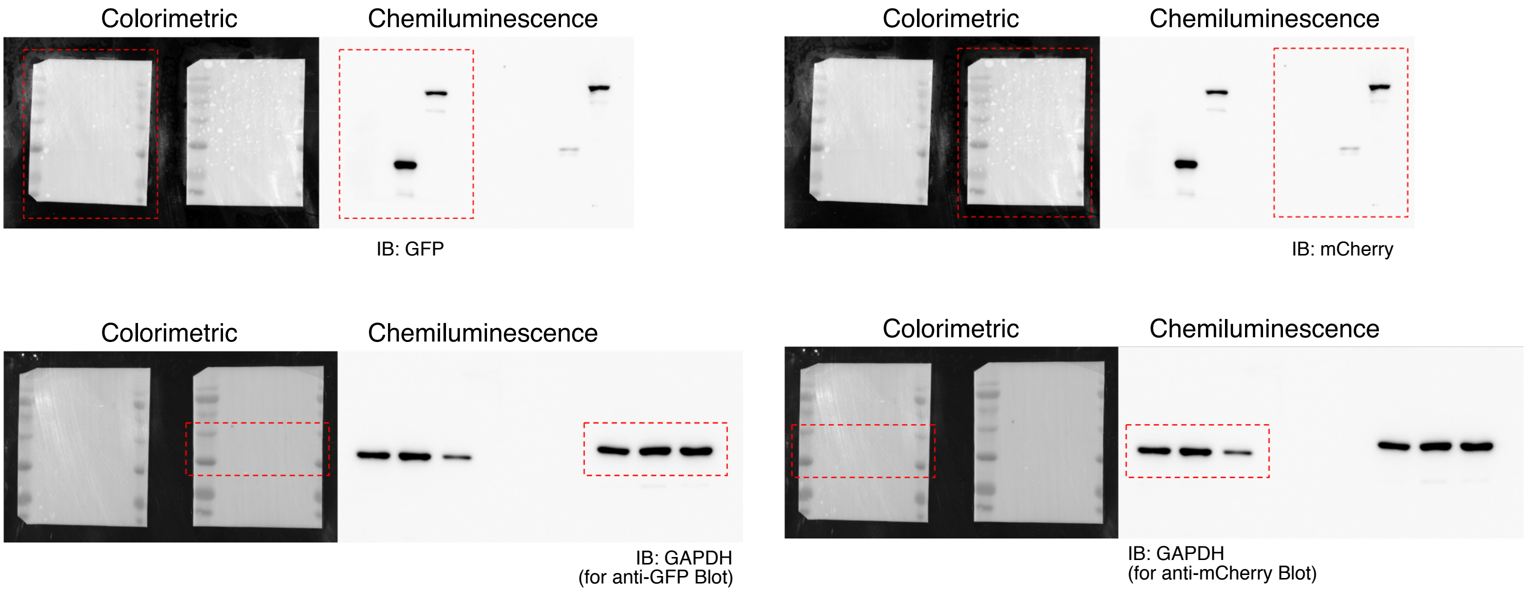

Supplemental Figure 1. Western blot analysis of GFP- and mCherry-PIPKH. Total protein extracts were prepared from Chinese hamster ovary cells expressing empty mCherry (mC) vector, empty GFP vector, mC-PIPKH or GFP-PIPKH. Samples were resolved by 10% SDS-PAGE and transferred to a 0.45 μm PVDF membrane. The membranes were blocked with 5% milk powder in PBS and probed with specific rat anti-mC and mouse anti-GFP antibodies. Bound antibody was detected using a horseradish peroxidase-linked anti-rat or anti-mouse IgG antibody. Glyceraldhyde-3-phosphate dehydrogenase (GAPDH) was used as load control. Images were captured using a BioRad ChemiDoc Touch Gel Imaging System. The molecular weights are indicated.

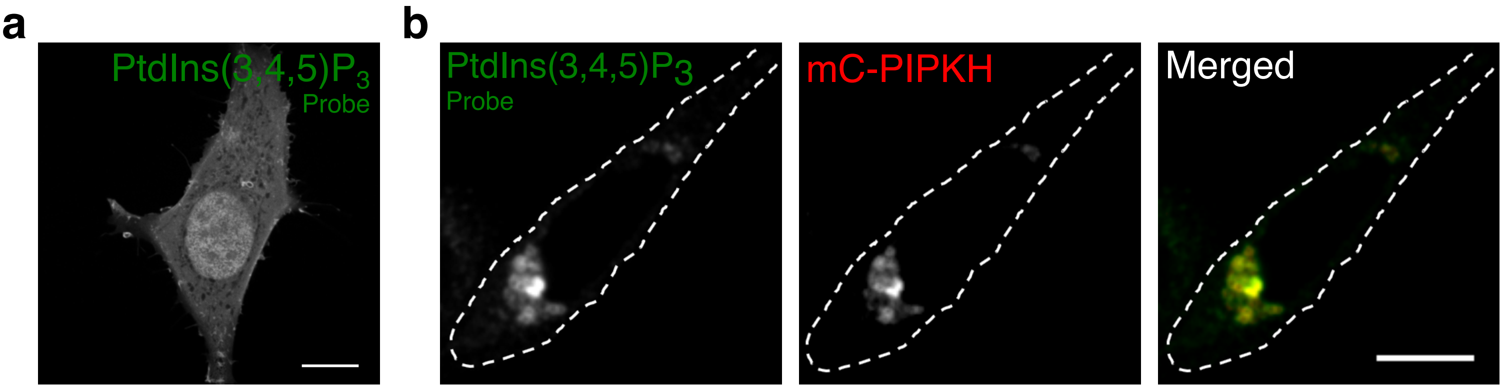

Supplemental Figure 2. Co-localization of PIPKH and the specific PtdIns(3,4,5)P_3 probe BTK-PH. (a) Chinese hamster ovary (CHO) cells were transiently transfected with GFP tagged Btk-PH (probe of PtdIns(3,4,5)P_3); (b) CHO cells co-transfected with GFP-Btk-PH and mCherry(mC)-tagged PIPKH. Images were acquired using spinning disc microscopy. Scale bars = 10 μm.
